# Use of exchange blood transfusion in the management of severe COVID-19 infection in pregnancy: experience from Lagos, Nigeria

**DOI:** 10.4314/ahs.v22i2.9

**Published:** 2022-06

**Authors:** Bosede Bukola Afolabi, Christian Chigozie Makwe, Kehinde Sharafadeen Okunade, Olanrewaju Nurudeen Akanmu, Iorhen Ephraim Akase, Oluwakemi Otokiti, Abiola Bolarinwa, Francis Ezenwankwo, Gabriel Olalekan Oyeleke, Beatrice Nkolika Ezenwa, Oluwaseun Martins-Akinlose, Titus Ogundare, Augustine Iloka, Alani Sulaimon Akanmu

**Affiliations:** 1 Department of Obstetrics and Gynaecology, College of Medicine, University of Lagos, Lagos, Nigeria; 2 Department of Obstetrics and Gynaecology, Lagos University Teaching Hospital Lagos, Lagos, Nigeria; 3 Department of Anaesthesia, College of Medicine, University of Lagos, Lagos, Nigeria; 4 Infectious Disease Unit, Department of Internal Medicine, College of Medicine, University of Lagos, Lagos, Nigeria; 5 Department of Haematology and Blood Transfusion, Lagos University Teaching Hospital, Lagos, Nigeria; 6 Department of Paediatrics, Lagos University Teaching Hospital, Lagos, Nigeria; 7 Neonatology Unit, Department of Paediatrics, College of Medicine University of Lagos, Lagos, Nigeria; 8 Department of Nursing Services, Lagos University Teaching Hospital, Lagos, Nigeria; 9 Department of Anaesthesia, Lagos University Teaching Hospital, Lagos, Nigeria; 10 Department of Haematology and Blood Transfusion, College of Medicine, University of Lagos, Lagos, Nigeria

**Keywords:** ARDS, COVID-19, EBT, Exchange blood transfusion and pregnancy, Nigeria

## Abstract

Coronavirus disease 2019 (COVID-19) presents with symptoms that may be mild or severe. The individual with the severe form of the disease usually presents with a constellation of respiratory symptoms typical of acute respiratory distress syndrome. In this report, we present our experience of the successful management of an oxygen-dependent pregnant woman with severe COVID-19 infection who had 2 sessions of partial exchange blood transfusion. We discussed the principles that informed this intervention and the need to adopt this novel approach in the care of severe COVID-19 infection.

## Introduction

Coronavirus disease 2019 (COVID-19) is a disease caused by a novel strain of coronavirus that causes severe acute respiratory syndrome (SARS-CoV-2). Individuals with a severe form of the disease may present with a constellation of respiratory symptoms typical of acute respiratory distress syndrome (ARDS) 1 requiring some form of ventilatory support. We report our experience with the use of partial exchange blood transfusion for the successful management of a pregnant woman with the severe COVID-19 infection who presented with features of acute respiratory distress syndrome ARDS).

## Case report

**Figure 1:**
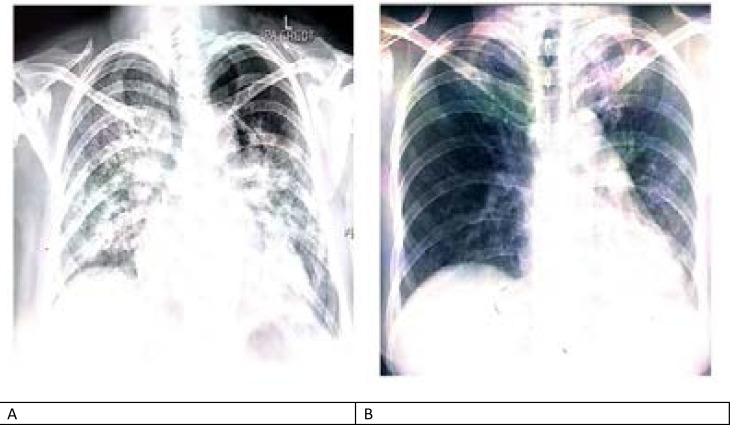
A – Chest Xray film at presentation showing multiple consolidations in the middle and lower zones predominantly and increased vascular markings B – Chest X-ray film at discharge showing subtle consolidations in the upper lung zones.

A 33-year-old gravida 3, para 2 Asian and a known asthmatic at 35 weeks gestation was referred to the Lagos University Teaching Hospital (LUTH). Prior to her referral, she had presented 3 days earlier at a private health facility with complaints of fever, cough, and breathing difficulty of one-day duration. She had no history of recent travel or contact with a confirmed case of COVID-19. She was managed for acute severe asthma without any improvement in her symptoms thus necessitating a chest radiograph that showed features suggestive of pneumonia and acute pulmonary oedema Figure 1A. She had reverse-transcription polymerase chain reaction (RT-PCR) of nasal and nasopharyngeal swabs and was confirmed to have the coronavirus disease 2019 (COVID-19) infection. On admission into the isolation unit of LUTH, her oxygen saturation (SpO2) was 76% on room air and 92% on 100% oxygen at 6 L/min via a non-rebreather mask. She was managed for acute severe asthma but her admission into the intensive care unit was not possible at this time due to the non-availability of bed spaces. The fetal heart rate was 152 beats per minute. However, on the second day of admission (DOA), she had a sudden deterioration of her clinical condition with SpO2 persistently below 88% while on 100% supplemental oxygen. Her laboratory results are as shown in Table 1.

**Table 1:** Laboratory parameters

Laboratory variables	Reference range	Day 2	Day 3	Day 4	Day 7	Day 8	Day 9	Day 12	Day 14
Sodium (mmol/L)	135 – 145	140	141			141		148‡	145
Potassium (mmol/L)	3.5 – 5.1	2.8^†^	3.6			3.3^†^		2.5^†^	3.9
Chloride (mmol/L)	98 – 110	102	107			89^†^		85^†^	81^†^
Bicarbonate (mmol/L)	22 – 30	20^†^	23			>30‡		>30‡	>30‡
Creatinine (µmol/L)	39 – 91	37^†^	40			31^†^		20^†^	40
Urea (mmol/L)	2.5 – 6.4	2.7	4.0			5.6		3.5	2.7
Anion gap (mmol/L)	3 – 15	18‡	11			22‡		14	4
Haemoglobin (g/dl)	12.0 – 16.0	10.8^†^	11.0^†^		11.6^†^		12.2	10.9^†^	11.1^†^
Haematocrit (%)	35 – 48	32.6^†^	36.9		36.7		37.6	38.5	38.6
Platelets (x 10^9^/L)	150 – 450	604‡	587‡		678‡		583‡	592‡	464‡
White cell count (x 10^9^/L)	4.0 – 11.0	11.9‡	18.1‡		15.6‡		15.7‡	14.3‡	9.9
Neutrophils (x 10^9^/L)	2.0 – 7.5	8.67‡	14.41‡		13.43‡		13.55‡	11.88‡	7.6‡
Lymphocytes (x 10^9^/L)	1.0 – 4.0	2.25	2.59		1.38		1.58	1.10	1.10
Monocytes (x 10^9^/L)	0.00 – 1.00	0.63	0.48		3.0		0.13	0.78	0.50
Eosinophils (x 10^9^/L)	0.00 – 0.40	0.33	0.54		0.46		0.40	0.47	0.74
Basophils (x 10^9^/L)	0.00 – 0.10	0.04	0.05		0.01		0.06	0.06	0.05
INR	0.9 – 1.3		0.85^†^		0.94			1.02	
ALP (U/L)	40 – 120		188‡					105	
ALT/SPGT (U/L)	10 – 40		31.1					25.4	
AST/SGOT (U/L)	10 – 42		65.0‡					34.1	
Gamma GT T(U/L)	7 -64		132‡					42.0	
PH			7.40	7.36	7.51				
PaCO_2_ (mmHg)			35	52	40				
PaO_2_ (mmHg)			96	49	-				
Lactate (mmol/L)			1.2	1.6	1.2				
FiO_2_			0.96	0.96	-				

‡ Elevated level beyond reference range

**Figure 2:**
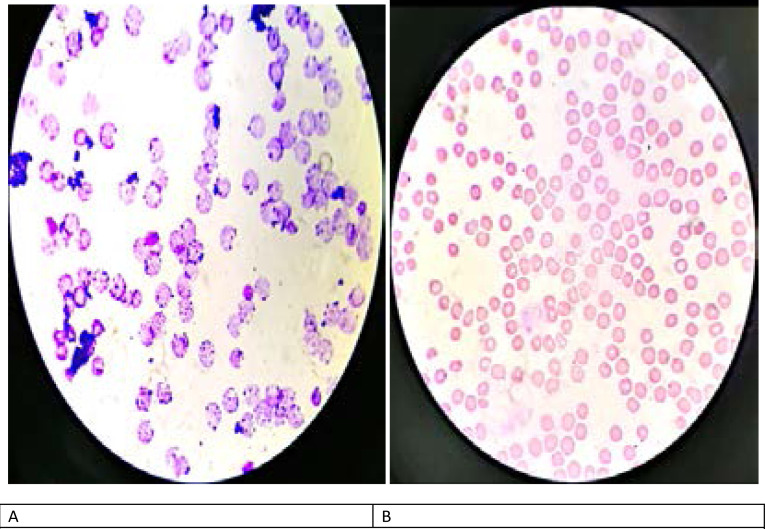
A – Patient red cells showing Heinz bodies pre-exchange transfusion X100 B – Patient red cells 4 days post-exchange transfusion (Heinz bodies hardly visible) X100

She subsequently had an emergency caesarean section performed under spinal anaesthesia and was delivered of a live female neonate weighing 3500 grams. Postoperatively, her clinical condition deteriorated further with worsening SpO_2_ (74 – 91%) despite being on continuous positive airway pressure (CPAP) with non-invasive ventilation delivering oxygen at 15 L/min via a non-rebreather mask. The peripheral blood film done on the 10^th^ DOA revealed Heinz bodies (methaemoglobin) in nearly 100% of her red blood cells Figure 2A. She subsequently had 2 sessions of EBT on the 12th DOA with a total of 4 units of whole blood transfused over 2 days. Following this, her condition improved significantly with a minimum SpO_2_ of 95% while on supplemental oxygen at 3–5 L/min. Her red cells now showed a significant reduction in methaemoglobin level Figure 2B. She was later transferred to the regular postnatal ward on the 16th DOA, having tested negative for COVID-19. She was discharged home with her baby after 22 DOA and then scheduled for a postnatal follow-up visit 2 weeks later. Figure 1B shows her chest X-ray at discharge.

## Discussion

Our knowledge regarding COVID-19 infection is still evolving at this time. The new thinking is that COVID-19 induced hypoxaemia as described in our patient may not solely be related to pulmonary pathology thus the premise for the use of EBT in our management. Oxygen is getting to the lungs but the red cells flowing in the alveolar capillary may have haemoglobin incapable of extracting and complexing with the oxygen. Wenzhong et al have shown that some SARS-CoV-2 regulatory proteins can impair haem synthesis and also cause dissociation of ferrous ion (Fe2+) from already formed haemoglobin, resulting in the inability of haemoglobin to transport oxygen to the tissues^2^. In particular, Coronavirus non-structural proteins OrfLab, ORF3a and the ORF10 were shown to be capable of complexing with the haem of the β -1 haemoglobin chain. This complex formation results in the dissociation of iron from the haem and the oxidation of Fe2+ to ferric ion (Fe3+) resulting in the formation of methaemoglobin^2^ which is incapable of carrying oxygen.

Thus, we premise that although our patient had a haemoglobin concentration optimal for pregnancy and puerperium, however, this only existed in a form incapable of reversible oxygen transfer. Recently, an alternative mechanism of SARS-Cov-2 induced haemoglobinopathy was also proposed – “the ferroptotic dysplasia”^3^. The viral protein is biodegraded by cellular proteases to smaller molecules which happen to have a remarkable resemblance to the master regulator of iron metabolism (hepcidin), thus providing the basis of the protein mimicry effect. This viral protein complexes with ferroportin and degrades it, resulting in impairment of iron release from cells into plasma (hypoferremia) and cellular iron accumulation. A situation is then created where there is a peripheral lack of iron, but the tissues are overloaded with iron. The excess cellular iron activates Fenton's chemistry with the generation of reactive oxygen species that progressively lead to cell death, a process referred to as ferroptosis^4^.

The ferroptotic events in erythroid blasts and red cells create a sideroblastic-like pathology with myelodysplastic features that should require the replacement of dysfunctional erythrocytes^3^. Finally, we also submit that the dramatic recovery observed in our patient after EBT may be due to the partial removal of inflammatory cytokines which is known to characterize severe COVID-19 infection. The virus induces severe inflammatory apoptosis (pyroptosis) resulting in the production of damaged cellular organelles resulting in Damaged-Associated-Molecular Patterns (DAMP)^5^. DAMP stimulates the macrophages and T-cells to produce chemokines and cytokines. These cytokines in a positive feedback mechanism caused the production of more cytokines (cytokine storm) which is associated with poor patient's survival^5^. Removal of these cytokines using an adsorbent agent in extracorporeal circulation has been shown to reverse severe disease in septic patients^6^. Therefore, an EBT may be an easier and cheaper alternative to extracorporeal blood purification in resource-limited settings such as ours.

## Conclusion

Exchange Blood transfusion appeared to be key to the reversal of oxygen dependency in a patient with severe COVID-19 pneumonia and this may have a role to play as an alternative to extracorporeal blood purification especially in resource-limited settings.
